# Plant root-microbe communication in shaping root microbiomes

**DOI:** 10.1007/s11103-015-0417-8

**Published:** 2016-01-04

**Authors:** Andrew Lareen, Frances Burton, Patrick Schäfer

**Affiliations:** School of Life Sciences, University of Warwick, Coventry, CV4 7AL UK; Warwick Integrative Synthetic Biology Centre, University of Warwick, Coventry, CV4 7AL UK

**Keywords:** Rhizosphere, Microbiome, Microbial communication, Plant root, Soil

## Abstract

A growing body of research is highlighting the impacts root-associated microbial communities can have on plant health and development. These impacts can include changes in yield quantity and quality, timing of key developmental stages and tolerance of biotic and abiotic stresses. With such a range of effects it is clear that understanding the factors that contribute to a plant-beneficial root microbiome may prove advantageous. Increasing demands for food by a growing human population increases the importance and urgency of understanding how microbiomes may be exploited to increase crop yields and reduce losses caused by disease. In addition, climate change effects may require novel approaches to overcoming abiotic stresses such as drought and salinity as well as new emerging diseases. This review discusses current knowledge on the formation and maintenance of root-associated microbial communities and plant–microbe interactions with a particular emphasis on the effect of microbe–microbe interactions on the shape of microbial communities at the root surface. Further, we discuss the potential for root microbiome modification to benefit agriculture and food production.

## Introduction

All eukaryotic organisms are influenced by complex interactions with microbial communities. The potential for gut microbiota to affect the health and nutritional status of host animals is well documented (Cummings and Macfarlane 1997; Hooper et al. 2002; Flint et al. 2012), and it is known that these microbial communities can be deliberately manipulated or inadvertently influenced through factors such as host diet (Turnbaugh et al. 2009), antibiotic use (Willing et al. 2011) and fecal transplants (Song et al. 2013). Like the animal gut, the primary organ for nutrient and water uptake in plants, the root system, is populated and surrounded by a complex microbial community referred to as the root microbiome (Hacquard et al. 2015). Interactions with the root microbiome have the potential to influence plant health and development (Berendson et al. 2012; Panke-Buisse et al. 2015). Direct interactions may range from parasitic (as is the case with soil-derived plant pathogens) through to mutualistic symbioses. Indirect effects are also of considerable importance. Microbes are key players in nutrient cycles and aid in nutrient acquisition (Mishra et al. 2012; Bulgarelli et al. 2013).

The importance of interactions between particular plants and specific microbial species is not a new concept. Beneficial symbiotic relationships such as between legumes and nitrogen-fixing microbial symbionts have been recognized for some time (Bergersen 1971), as too have the detrimental effects of plant pathogens on crops (Oerke 2006). Aside from the effects of specific pathogens and symbionts on plant health, recent research has indicated that the composition of microbial communities at roots, the so called root microbiome, can have significant impacts both on plant development and their stress tolerance (Mendes et al. 2011; Panke-Buisse et al. 2015). Some consider the root microbiome a “secondary genome” that provides host plants with microbe-derived compounds and traits (Berendson et al. 2012; Rout and Southworth 2013).

The root microbiome is recruited from a diverse range of microbes present in the surrounding bulk soil (soil biome outside the rhizosphere). The emergence of dominant groups in the rhizosphere from this soil biome can have major implications for resident plant species. While soil biomes are undoubtedly a key determinant of root microbiome composition, research has demonstrated that host genotype also influences the overall composition of these communities (Badri et al. 2013; Bulgarelli et al. 2012, 2015). As it is largely plant-derived exudates and substrates that provide the nutrients and physical niches of the rhizosphere, it perhaps makes evolutionary sense that plants should have adapted to influence this ecosystem to their benefit. However, opinion is divided as to whether it is edaphic factors or selection by plants that are the greatest determinant of root microbiome composition. What is clear however is that both edaphic and host-plant factors exert strong influences over its formation (Chaparro et al. 2012; Hacquard et al. 2015). Root-associated microbial communities have been the focus of much research. However, the factors and multipartite interactions that can lead to changes in root microbiome structure, and, hence, affect plant health and development, are highly complex, dynamic and not fully understood. Exploiting the beneficial potential of the root microbiome can provide sustainable solutions in raising agricultural crop production (Philippot et al. 2013). In particular, diseases caused by soil-borne microbes have a major negative impact on global crop productivity and account for major losses in wheat, rice, potato, maize and soybean (Oerke 2006; Raajmakers et al. 2008; Alexandratos and Bruinsma 2012). In the context of increased demand for food by an expanding human population, coupled with reductions in cultivable land and agricultural productivity due to development and climate change effects (Alexandratos and Bruinsma 2012), understanding the interaction of plants with microbial communities and development of methods for manipulation of microbiome composition to encourage plant-beneficial relationships is increasingly relevant. This review provides an overview of what is known about microbial community dynamics, with a detailed focus on the potential for manipulation of the root microbiome to increase crop yields and reduce losses to biotic and abiotic stresses. In addition to introducing briefly the effect of plants and soil on microbome composition, we will particularly discuss the effect of microbial interactions on microbiome composition and dynamics.

## Plant and soil-derived determinants affecting microbial root communities

Soil type and plant roots can determine the composition of microbial communities associated with roots though their quantitative contribution in influencing rhizosphere communities (rhizobiome) is unclear. The effect of soil and plants on the composition of rhizosphere communities has been excellently reviewed recently (Berg and Smalla 2009; Philippot et al. 2013; Bulgarelli et al. 2013, 2015) and therefore only an overview is provided here. Soils can vary in pH, structure, texture, organic matter content, microaggregate stability and the availability of nutrients. These physico-chemical properties of soils can directly select for specific microbes by creating niche environments that benefit certain types of microbes and influence the availability of plant root exudates affecting microbial recruitment by the plant. For instance, soil pH and nutrient availability (e.g. carbon, nitrogen, phosphate) have been found to affect the abundance of crop pathogenic bacteria, fungi and nematodes as well as beneficial microbes (Höper et al. 1995; Duffy et al. 1997; Lacey and Wilson 2001; Rasmussen et al. 2002; Rimé et al. 2003; Hamel et al. 2005; Rotenberg et al. 2005; Toljander et al. 2008; Dumbrell et al. 2010). In most extreme cases soil characteristic might result in soil type-specific composition of rhizosphere microbial communities (Garbeva et al. 2004). Consistent with this, Gelsomino et al. (1999) have shown that the structure of bacterial communities was similar in soils of the same type rather than geographical location and Latour et al. (1996) observed that soil type affected the diversity of *Pseudomonas* spp. associated with flax and tomato plants. This indicates that soil type and soil characteristics can influence which microbes dominate the rhizosphere, and that different types of soils can harbour diverse microbial communities.

Significant effects on the composition of rhizosphere communities have been assigned to soil types and plant species (Chiarini et al. 1998a, b; Grayston et al. 1998; Buyer et al. 1999; Dalmastri et al. 1999; Miethling et al. 2000; Smalla et al. 2001; da Silva et al. 2003; Rasche et al. 2006; Bulgarelli et al. 2012; Lundberg et al. 2012; Peiffer et al. 2013; Tkacz et al. 2015) suggesting a hierarchic contribution of soil and plant species on microbial communities (Bulgarelli et al. 2013; Philippot et al. 2013; Schlaeppi et al. 2014). Whereas physico-chemical properties of soil types determine the composition of soil biomes, plant root exudates can create an environment at the rhizosphere that gradually alters the soil biome to favor the establishment of a rhizobiome. These exudates together with the root immune system would finally select for those microbes that due to further adaptation have evolved mechanisms to colonise the root rhizoplane and/or inner root tissue (endosphere). Endophytes or colonisers of the rhizoplane can have detrimental or beneficial effects on plant species and resulting changes in the structure of the plant community would feedback in the composition of the rhizobiome (Bever et al. 2012). In such a model, in addition to soil properties, plant exudates and microbial activities would determine the magnitude of biome conversion (Bever et al. 2012; Bakker et al. 2013; Bulgarelli et al. 2013; Philippot et al. 2013). Further, recent studies highlighted the significance of hormones involved in plant immunity, and especially salicylic acid, in shaping the root microbiome (Lebeis et al. 2015).

Plant roots exude a variety of compounds into the soil, including carbohydrates, amino acids and organic acids (Jones 1998; Bais et al. 2006) by diffusion, ion channels and vesicular transport (Bertin et al. 2003). These compounds alter soil chemistry and provide nutrient sources for microbes in the rhizosphere (Lynch and Whipps 1990; Bardgett et al. 1998; Bever et al. 2012; Miransari 2013). Studies with Arabidopsis, barley, maize, potato or sugarcane revealed, in addition to a soil-dependent variation, a genotype-dependent variation in the composition of the rhizosphere community (Rasche et al. 2006; Bulgarelli et al. 2012; Lundberg et al. 2012; Peiffer et al. 2013; Bulgarelli et al. 2015; Lebeis et al. 2015; Yeoh et al. 2015). These results are intriguing as it suggests a targeted restructuring of the rhizobiota by plants to serve their own benefits. Further, potato development slightly but significantly affected the rhizosphere community composition (Rasche et al. 2006), which is in accordance to Chaparro et al. (2014), who observed plant development-dependent changes in the composition of rhizobiomes that were associated with slight alterations in its meta-transcriptome. These findings might indicate a development-specific release of root exudates to establish microbiota activities that can enhance plant fitness. Consistent with this, wild oat roots showed root zone-dependent difference in microbial communities with higher bacterial cell counts in the root tip and root hair zone as compared to bulk soil (DeAngelis et al. 2009). Consequently plants species can have different microbial communities associated with their roots. This can lead to the selective enrichment of specific microbes along the root axis in the rhizosphere and support overall plant health and development (Berendsen et al. 2012). Moreover, it might provide a source to use such plant–microbe interactions to identify heritable traits to improve crop productivity (Mendes et al. 2013; Peiffer et al. 2013) or to select for microbiomes that can improve crop traits as has been reported for the genotype-driven selection for microbiomes that altered flowering in *Arabidopsis thaliana* and *Brassica rapa* (Panke-Buisse et al. 2015).

Plants apparently employ and adjust root exudate composition. This ability varies among plant species and genotype and further depends on age, nutritional status and stress exposure (Haichar et al. 2008; Compant et al. 2010; Bever et al. 2012; Pérez-Jaramillo et al. 2015; Philippot et al. 2013). The discovery that different plant species can have different microbial communities associated with their roots indicates that particular types of exudates attract or repel specific microbes (Grayston et al. 1998; Bertin et al. 2003; Kumar et al. 2007; Marschner et al. 2011; Berendsen et al. 2012). Importantly, root exudates can modify the root microbiome in absence of the plant. Badri et al. (2013) described the application of natural blends of phytochemicals obtained from *Arabidopsis* to a soil, and monitoring of subsequent changes to the bacterial community via 16S rRNA gene pyrosequencing. They demonstrated that phytochemicals, predominantly phenolic-related compounds, modify the bacterial community by stimulating or inhibiting different community members. This highlights the agricultural potential of plant-derived compounds in inducing plant-beneficial microbial communities in soils. It might represent a strategy to enhance plant protection against pathogens or improve nutrient and water acquisition abilities of plants. Especially as we know that plants use root exudates to attract mutualistic microbes that can improve their nutrient supply (Parniske 2008; Marschner et al. 2011; Oldroyd 2013). Under iron deprivation, plants have evolved two mechanisms to increase iron solubility in the rhizosphere. Both strategies convert inorganic Fe^III^ into Fe^II^, which can be taken up readily by plants. Strategy I involves a plasma membrane bound reductase to convert Fe^III^ to accessible Fe^II^. Strategy II is mediated by the release of Fe^III^ chelating phyto-siderophores (Römheld 1987). Microbes in the rhizosphere also produce siderophores to increase the amount of soluble iron for uptake. Plants profit from this increased Fe^II^ availability and therefore select for these advantageous microbes through their root exudes in order to enhance iron availability (Hartmann et al. 2009; Carvalhais et al. 2013). To improve phosphate and nitrogen supply, plant roots release strigolactones to attract mycorrhiza (Akiyama et al. 2005) and legumes secrete specific combinations of flavonoids to establish symbioses with nitrogen-fixing rhizobia, respectively (Bertin et al. 2003; Hassan and Mathesius 2011). A well-studied example is soybeans that secrete isoflavones in order to host the endosymbiotic nitrogen-fixing bacterium *Bradyrhizobium japonicum* (Morris et al. 1998). Rudrappa et al. (2008) demonstrated selective recruitment by *Arabidopsis thaliana* of the beneficial rhizobacterium *Bacillus subtilis* FB17 when challenged with foliar pathogen *Pseudomonas syringae* pv *tomato* (*Pst* DC3000). Recruitment is mediated by l-malic acid, a tricarboxylic acid cycle intermediate secreted by roots in response to *Pst* DC3000 infection of foliage. Transcriptome analyses revealed that the interaction with *B. subtilis* FB17 systematically altered the expression of Arabidopsis genes involved in auxin regulation, metabolism, defence and stress responses as well as cell wall modification (Lakshmanan et al. 2012). Increased populations of beneficial *B. subtilis* at the root in response to aphid attack of foliage have also been observed in *Capsicum anuum*, correlated with reduced populations of the pathogen *Ralstonia solanacearum* (Lee et al. 2012). These studies suggest that, in response to pathogen or herbivore attack, plants are able to specifically signal and recruit beneficial microbes and that these symbioses result in a beneficial reprogramming of the host. It is also likely that these recruited microbes compete with other, potentially pathogenic, soil microbes. In this respect it is, however, important to note that such communication strategies are prone to highjacking by parasitic organisms. The oomycete pathogen *Phytophthora sojae*, for example, is attracted by isoflavones and exploits this communication system to find host plants (Morris et al. 1998; Subramanian et al. 2007; Cameron et al. 2013) and the parasitic weed Striga perceives strigolactones to find and colonise host plants (e.g. wheat) (Scholes and Press 2008).

Despite the potential exploitation by pathogenic and opportunistic microbes, creating a rhizosphere milieu might allow plants to establish robust beneficial microbial communities to obtain a variety of benefits and enhance their ecological competitiveness. But the key might be to recruit a high diversity of beneficial microbes to sustain nutrient supply and receive protection against pathogens and opportunists. Therefore, understanding microbe–microbe dynamics and their effects on the composition of microbial communities is essential to identify microbial determinants that shape microbial communities. This knowledge can provide solutions to create beneficial microbiomes as apparently present in suppressive soils (see below) that can sustainably enhance crop production.

## How do microbe–microbe interactions affect soil communities?

The rhizosphere as a plant nutrient enriched site is a highly competitive environment for microbes. Microbes produce secondary metabolites to outcompete competitors that occupy similar niches and to establish at the rhizosphere or inside roots (Thomashow and Weller 1988; van Loon and Bakker 2006; Pierson and Pierson 2010; Kim et al. 2011). These metabolites include antibiotics, toxins, lytic enzymes and siderophores (Bais et al. 2006). Some rhizosphere microbes possess large gene clusters involved in detoxification, production/release of antibiotics and siderophores, including *Bacillus amyloliquefaciens* (Chen et al. 2007) and *Pseudomonas fluorescens* (Paulsen et al. 2005). Common antibiotic compounds secreted by microbes include 2,4-diacetylphloroglucinol (DAPG), hydrogen cyanide, oomycin A, and phenazine (van Loon and Bakker 2006). These antibiotics are important for the suppression of pathogens in soils (Raaijmakers and Mazzola 2012), e.g. phenazines produced by *Pseudomonas chlororaphis* against *Fusarium oxysporum* (Pierson and Pierson 2010; Thomashow and Weller 1988; Kim et al. 2011). In addition, lower concentrations of antibiotic compounds released by some microbes have led to the suggestion that the primary function of some of these molecules is in communication rather than inhibition or exclusion of competitors (Aminov 2009). This range of functions in the soil suggests antimicrobial compounds as key in establishing microbial communities in the rhizosphere. As one would anticipate under such conditions, a wide array of antibiotic resistance genes (ARG) is to be found in soil microbiomes. The potential for horizontal gene transfer of ARG may have important implications for agriculture in the future, but also for broader human health, should human-pathogenic microbes be present in the soil. A detailed account of antibiotic resistance reservoirs has recently been reviewed (Cytryn 2013).

In addition to antibiotic compounds, microbes produce secondary metabolites to alter plant signalling and metabolism (Brazelton et al. 2008; Constacurta and Vanderleyden 1995; Kim et al. 2011) in order to receive nutrients (Prikyrl et al. 1985). This microbial reprogramming of the plant can alter the composition of root exudates and induce the release of more favourable exudates, which may lead to a selective enrichment of respective microbes in the rhizosphere (Prikyrl et al. 1985; Bulgarelli et al. 2013). This suggests that antimicrobial compounds and secondary metabolites are important factors in establishing microbial communities in the rhizosphere, which aid in competitive niche exclusion. Competitiveness as a prerequisite for the establishment and dominance of communities requires a coordinated communication between microbes as well as the perception and translation of environmental signals.

### Bacterial cell-to-cell communication

Research in recent decades has highlighted the degree to which bacteria can communicate, and the importance of this to their survival and competitiveness (Atkinson and Williams 2009). This can result in outcomes as diverse as inhibition of competitors through to cooperative behaviour that provides both individual and group level benefits (Atkinson and Williams 2009; Rocha et al. 2012; An et al. 2014). The underlying communication between bacterial microbes is undoubtedly an important factor in root microbiome dynamics (Yajima 2014; Atkinson and Williams 2009).

Communication between bacterial cells is reliant upon the synthesis and diffusion of signal molecules that are subsequently perceived by other community members. Upon perception, signal molecules induce changes in gene transcription thereby altering the physiology and activity of the recipient (Atkinson and Williams 2009). Communication is therefore of importance in the regulation of bacterial functions that require coordination between community members. These include biofilm formation, adhesion and motility (Sperandio et al. 2002; Chu et al. 2011). Signal molecule-mediated communication has also been associated with changes in metabolic rate (An et al. 2014), control of virulence-associated factors (Sperandio et al. 2002; Chu et al. 2011) and propagation (Rocha et al. 2012). Regulation of these aspects of bacterial behaviour is often correlated to population density. This type of density-dependent stimulus and response system is known as quorum sensing (QS) (Fuqua et al. 1994; Miller and Bassler 2001; Atkinson and Williams 2009; An et al. 2014). It has become increasingly clear that signal molecule-mediated communication is not restricted to related prokaryotic organisms. Signal molecules can be intercepted and acted upon by non-related prokaryotes, and also be used to the competitive advantage of the producer by modifying the behaviour of unrelated recipients (Atkinson and Williams 2009). In addition, signals can be degraded by competing microbes to the detriment of the producer (Dong et al. 2000; Molina et al. 2003; Uroz et al. 2003; Dong et al. 2004; Morello et al. 2004; Newton and Fray 2004). There are a variety of communication systems utilized by prokaryotes. These differ in the type of chemical compounds produced as signal molecules and the molecular machinery used to receive and integrate the signals. Signal molecules include *N*-acylhomoserine lactones (AHL), a type utilised by the majority of Gram negative bacterial species in QS regulation of activities such as biofilm formation, bioluminescence and secretion of virulence factors. In addition, Autoinducer-2 (AI-2), a QS pheromone regarded as conserved amongst Gram negative bacteria and a range of small peptides or post-translationally modified peptides that comprise the majority of Gram positive QS molecules (Yajima 2014).

### Fungal cell-to-cell communication

Signal-mediated cell-to-cell communication has been demonstrated in fungi as well. As with bacteria, this communication is reliant upon synthesis and diffusion of signal molecules that are perceived and integrated by the recipient. Density-dependent regulation of activities that require coordination at the population level has also been demonstrated. QS molecules such as farnesol and tyrosol have been implicated in the regulation of biofilm formation, morphogenesis and drug resistance (Chen et al. 2004; Enjalbert and Whiteway 2005; Alburquerque and Casadevall 2012). Phenylethanol and tryptophol have been shown to regulate morphogenesis in *Saccharomyces cerevisiae* in a QS system that is both density-dependent and responsive to environmental nutrition status (Chen and Fink 2006). While these examples are taken from organisms that are not major components of soil microbial communities, they do serve as indicators that signal molecule-mediated communication systems are ubiquitous and of importance in the fungal kingdom.

### Cross-domain and cross-kingdom communication

**Fig. 1:**
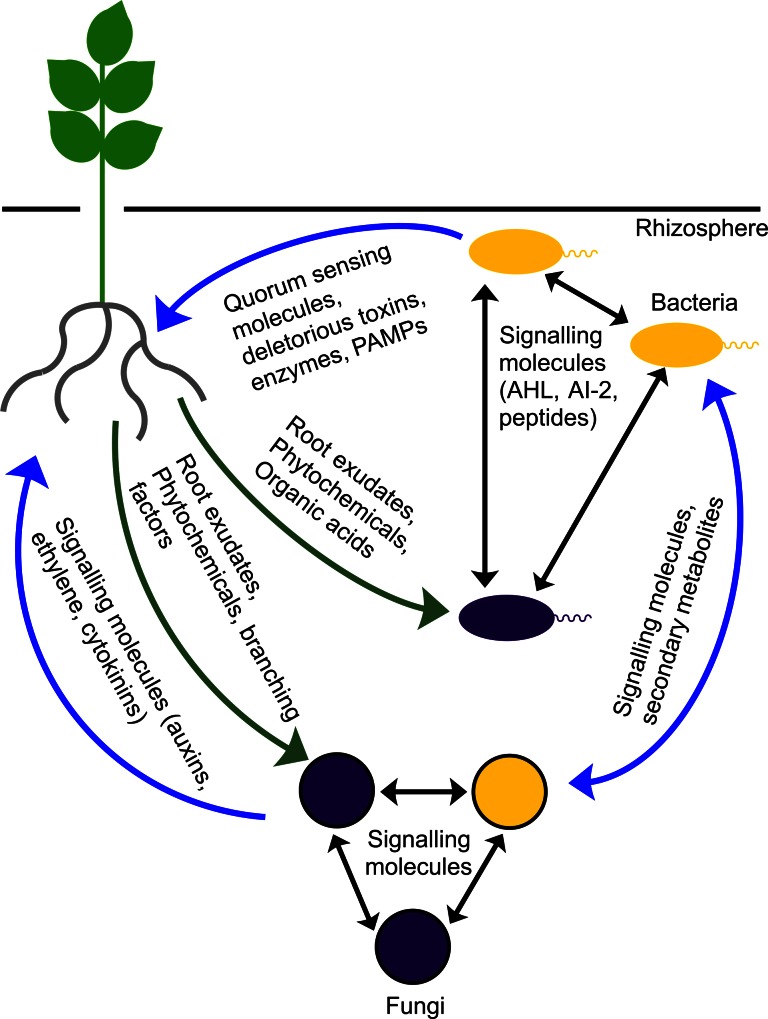
Schematic overview of interactions between plants, fungi and bacteria in the rhizosphere. Microbial communities in the rhizosphere communicate with each other and the plant root using a variety of mechanisms, including bacterial AHLs (N-acylhomoserine lactones) and AI-2 (Autoinducer-2). This can directly influence the composition of microbial communities, and in some cases lead to improved plant health when plant roots establish beneficial interactions with root microbes

In addition to intra-domain signal molecule-mediated communication it is increasingly evident that signal molecules can traverse the domain divide. Underlying communication strategies are utilised by plants, fungi and bacteria in the rhizosphere (Fig. 1). Bacteria employ signalling molecules to elicit responses in eukaryotes such as plants and fungi. Volatile organic compounds (VOCs) produced by plant growth-promoting rhizobacteria (PGPR), for example, promote growth in *Arabidopsis thaliana* (Ryu et al. 2003) and initiate induced systemic resistance (ISR) in plants, thus stimulating expression of defence genes that can be effective against fungi, bacteria, oomycetes and viruses (Heil and Bostock 2002; Zhang et al. 2002). While known to regulate bacterial activities in a density-dependent fashion, QS molecules also elicit a range of plant-beneficial responses in host plants. These include plant “priming”, in which exposure to quorum signaling molecules primes the plant to respond more robustly and rapidly to biotic challenges (Schenk and Schikora 2015). Schuhegger et al. (2006) further demonstrated that exposure to AHL produced by *Serratia liquefaciens* MG1 and *Pseudomonas putida* IsoF increased systemic resistance of tomato plants against the fungal foliar pathogen *Alternaria alternate* by inducing ethylene and salicylic acid-dependent defence genes (Schuhegger et al. 2006). The AHL N-3-oxo-tetradecanoyl-L-homoserine lactone also supports pathogen defense in Arabidopsis by promoting enhanced deposition of callose, accumulation of phenolic compounds, lignification of cell walls and stomatal closure in response to *Pseudomaonas syringae* infection (Schenk et al. 2014). Importantly, these AHL activities are associated with an increase in salicylic acid and oxylipin levels. The stimulation of plant hormone activities is further used by some bacterial and fungal strains isolated from the rhizosphere of maize and bean that are capable of producing auxin (Prikyrl et al. 1985). This may induce a redirection of nutrient flow by the plant towards the site of colonisation, thereby benefiting the producers. Alternatively it may stimulate carbohydrate release from the plant cell wall (Kim et al. 2011). Similarly, indole, an important bacterial signalling molecule involved in a range of cooperative activities such as production of virulence factors and biofilm formation, can manipulate plant root development through interference with auxin signalling (Bailly et al. 2014).

Bacteria-derived molecules may also modulate fungal development under certain conditions. It has recently been demonstrated that bacterial metabolites can act as sporulation signals to filamentous fungi in co-culture biofilms. Redox-active secondary metabolites secreted by *Pseudomonas aeruginosa* shifted *Aspergillus fumigatus* development from weak vegetative growth to induced asexual sporulation (Zheng et al. 2015). Finally, our growing understanding of the processes involved in the early stages of legume-rhizobia symbioses and root mycorrhization indicate inter-domain communication for their successful establishment (Janczarek et al. 2015; Kosuta et al. 2003; Oláh et al. 2005; Sun et al. 2015). These studies suggest a potential for modification of plant and fungal development through application of microbial species or derivative metabolites.

## Effects of multipartite interactions on plant performance

In the past, research merely focused on pair-wise interactions between species of bacteria, fungi and plants. However, a growing body of research is highlighting the importance of multipartite interactions to impacts on host plants. Balbontin et al. (2014) found co-inoculation of maize plants with *Salmonella enterica* serovar Typhimurium and *Aspergillus niger* caused a greater decrease in plant height than inoculation with either the bacteria or fungus alone. This is suggestive of additive or synergistic effects of the inoculants on growth suppression. They further observed that the association between *A. niger* and *S. enterica* is mutualistic. *A. niger* promoted growth of the bacteria, while bacterial biofilms afforded protection to the fungus from the anti-fungal agent cycloheximide. Similarly, co-inoculation of the prairie legume *Amorpha canescens* with arbuscular mycorrhizal fungi (AMF) and rhizobial bacteria produced greater increases in plant biomass than inoculation with AMF or rhizobia alone. These increases were also found to be higher than predicted additive effects based on the performance of AMF and rhizobia strains when applied as separate inoculants (Larimer et al. 2014), suggestive of synergistic effects of rhizobia and AMF on *A. canescens* growth. The greater plant–beneficial effects of tripartite symbioses when compared to single microbe-plant interactions are further supported by van der Heijden et al. (2015). Legume seedlings obtained a 15-fold higher productivity when forming an association with both an AMF and a nitrogen-fixing rhizobia, compared to that achieved with either AMF or rhizobia alone. In terms of plant beneficial effects, the results of the aforementioned studies provided a more positive picture of multipartite interactions than a meta-analysis of the interactive effects of plant-microbial symbionts (Larimer et al. 2010). The analysed data did not support the hypothesis that AMF and rhizobia should act synergistically in improving plant performance. They did however find that the negative impacts on plants of antagonistic fungal endophytes were alleviated by the association with AMF. These investigations and meta-analyses highlight the need for increased research into the effects of multipartite interactions on plant health, and the importance of this to strategies aimed at increasing crop performance through manipulation of microbial communities in the rhizosphere.

## Stable beneficial communities in agricultural soils

Understanding the principals of microbe–microbe and plant–microbe communication provides the potential to generate beneficial microbial communities in agricultural soils. The question is whether this is feasible and whether such microbial communities would be stable. Natural disease suppressive soils indicate the existence of merely beneficial soils that allow increased crop yield and productivity. These soils are defined as “soils in which the pathogen does not establish or persist, establishes but causes little or no damage, or establishes and causes disease for a while but thereafter the disease is less severe, although the pathogen may persist in the soil” (Baker and Cook 1974). Plants grown in these soils experience lower disease severity and incidence when compared to surrounding soils, making the causal underlying mechanism of this phenomenon intriguing for enhancing food security.

It has been suggested that stable populations of beneficial microbes that are selectively recruited and maintained in the rhizosphere by the plant, subdue pathogens through secretion of secondary metabolites (Doornbos and Van Loon 2012). This ultimately leads to disease suppression and perhaps full or partial exclusion of the pathogen from the soil. There are two classifications of disease suppressive soils: general and specific suppression. In both cases pathogen persistence and virulence in the soil is severely inhibited (Janvier et al. 2007). General suppression is when microbial activities in the rhizosphere suppress pathogen growth. This could be induced through the addition of organic matter to the soil, which increases microbial activity and competition, thus resulting in disease suppression. Specific suppression occurs when specific microbes antagonise the pathogen, causing soils to suppress diseases (Weller et al. 2002; Berendsen et al. 2012). For instance some microbes are able to suppress the soil-borne pathogen *Rhizoctonia solani* (Mendes et al. 2011), including *Pseudomonas* spp. which secrete phenazime-1-carboxylic acid and 2,4-DAPG (Raaijmakers et al. 1997). The production of lipoproteins by *Pseudomonas* and *Bacillus* spp. can also inhibit growth of a wide range of pathogens (Raaijmakers et al. 2010; Watrous et al. 2012; Zachow et al. 2015).

Further insights into the microbial and functional nature of suppressive soils came from studies of take-all decline (TAD). TAD is defined as the decrease in prevalence and disease severity in wheat and other susceptible hosts due to pathogen suppression in the rhizosphere (Weller et al. 2002). TAD occurs globally, despite differences in soil type, climates and agricultural practices. Disease suppression against the take all soil-borne plant disease caused by the fungus *Gaeumannomyces graminis* var. *tritici* in wheat is induced when susceptible crops are grown in monoculture after at least one severe outbreak of disease. Disease suppression falters when monoculture is no longer used or a non-host plant is introduced into the field indicating that suppression is not naturally associated with the soil, and that several generations of plants affect the establishment of these disease suppressive microbes (Cook 1993; Weller et al. 2002). One particular group of bacteria that have been implicated in TAD development are *Pseudomonas* spp. that synthesise 2,4-DAPG (Weller et al. 2002). 2,4-DAPG inhibits *Gaeumannomyces graminis* var. *tritici* growth by impairing ATP synthesis, as it disrupts the proton gradient across the mitochondrial membrane (Troppens et al. 2013). The concentration of 2,4-DAPG-producing bacteria and the severity of take-all are inversely proportional, and TAD is eliminated when soil containing 2,4-DAPG-producing bacteria is pasteurised (Raaijmakers and Weller 1998). This indicates that these *Pseudomonas* spp. can significantly contribute to the take-all disease in soils. These antagonistic *Pseudomonas* bacteria probably become selected and enriched in the rhizosphere during wheat monoculture, leading to the establishment of TAD. Moreover, these findings suggest the feasibility to alter microbial communities and to generate customised microbial communities. In support of this, disease suppression has been induced by inoculating soil with beneficial microbes. For instance, *Phyllachora huberi*, causing black crust on the leaves of *Hevea brasiliensis*, was suppressed by applying *Cylindosporum concentricum* and *Dicyma pulvinata* inoculum to soils (Sutton and Peng 1993; Cook 1993).

## Customised adjustment of soil microbial communities

The study of suppressive soils highlights the potential of customised adjustment of microbial communities to bring benefits to crop production in terms of plant growth and resistance to biotic and perhaps abiotic challenges. Utilising microbes in agricultural settings is not a new concept. Commercially available entomopathogenic *Bacillus thuringiensis* strains are widely used to protect agricultural crops from specific insect pests of foliage, while microbial inoculants are available for soil enrichment (Sanchis and Bourguet 2008). Research into the effects of deliberate and specific application of soil-borne plant-beneficial microbes has also been conducted. Mixtures of strains of the PGPRs *Bacillus pumilus*, *Bacillus subtilis* and *Curtobacterium flaccumfaciens* applied to cucumber seeds enhanced biological control of several cucumber pathogens in addition to increasing plant growth (Raupach and Kloepper 1998). Plant growth promoting *Azospirillum* species have also long been recognised for the benefits they can bring to host plants (Bashan and Holguin 1997; Veresoglou and Menexes 2010), and are used in agricultural settings for biofertilisation of many crops (Namvar and Khandan 2015). A meta-analysis on 59 investigations related to the effects of *Azospirillum* inoculation on seed yield and above-ground dry weight in wheat found a mean increase of 8.9 and 17.8 % respectively (Veresoglou and Menexes 2010). More recent studies demonstrated increased grain yield and oil content in rapeseed (*Brassica napus*) following application of inoculum comprising *Azospirillum* spp. and *Azotobacter* spp. (Namvar and Khandan 2015). These effects have variously been attributed to indole acetic acid production, gibberellins, a variety of polyamines and amino acids, and increased nutrient availability to plants (Thuler et al. 2003; Bashan and de Bashan 2010; Veresoglou and Menexes 2010; Namvar and Khandan 2015). While the benefits to plants of *Azospirillum* inoculation is well supported by some studies, further research is required to fully understand how *Azospirillum* may persist in the soil post inoculation, which is important in the context of large scale crop production. Herschkovitz et al. (2005), for instance, revealed that inoculation of *Zea mays* roots with the PGPR *Azospirillum brasiliense* had no effect on bacterial community structure. *Azospirillum* is certainly only one example of microbes that may struggle to compete in established microbial communities under certain field conditions. In order to establish applied strategies to improve microbial persistence, we need to understand which biotic (e.g. plant age, genotype, microbe–microbe interactions) and abiotic factors (e.g. nutrient and water availability, soil type, soil physics) determine microbial community dynamics and composition under field conditions.

In addition to beneficial bacteria, the importance of fungal symbionts to many plant species is well documented. In particular AMF are recognized for their ability to increase host access to mineral nutrients, predominantly phosphate. Their presence has also been associated with reductions in bacterial foliar pathogens (for review see; Parniske 2008). Many non-AMF strains can also bring benefits to plants. Strains of the endophyte *T. harzianum* are already commercially available as fungicides and recent experiments have highlighted the benefits of *T. harzianum* soil enrichment. Application of *T. harzianum*-enriched biofertiliser to tomato plants allowed chemical fertiliser input to be reduced by 25 % with no reduction in yield (Cai et al. 2014). This suggests *T. harzianum* has the potential to bring financial benefits for producers while reducing the environmental harm of chemical fertilizer application. Earlier work demonstrated supplementation of continuously cropped cucumber soil with *T. harzianum*-enriched bioorganic fertilizer increased microbial diversity. This was associated with reductions in severity of *Fusarium* wilt disease (Chen et al. 2012). Of current interest to several groups is the Sebacinales fungus *Piriformospora indica*. *P. indica* is an endophytic fungus able to infect the roots of a wide range of plant species (Oelmüller et al. 2009; Weiß et al. 2011). In addition, endophytic members of the Sebacinales are ubiquitous in a range of ecosystems (Weiß et al. 2011), indicative of competitive life strategies that potentially involve influence over microbial community dynamics at the rhizosphere. Infested plants have been observed to produce higher yields and display increased tolerance of biotic and abiotic stresses when compared to controls (Waller et al. 2005). Increases in plant growth may be attributable to an increased ability to acquire nutrients, in particular phosphorous, in the presence of *P. indica* (Yadav et al. 2010; Ghanem et al. 2014). Interestingly, studies involving co-inoculation of *Cicer arietinum* (chickpea) with *P. indica* and the PGPR *Pseudomonas striata* found the presence of *P. indica* resulted in short term increases in *P. striata* in the rhizosphere (Meena et al. 2010). Plant beneficial effects were only observed with co-inoculation of the two microbes, suggesting synergistic effects on increases in *P. striata* population and plant biomass (Meena et al. 2010). Potential synergism has also been demonstrated in challenge of *C. arietinum* with *Macrophomina phaseolina* (root-rot fungus) and *Meloidogyne incognita* (root-knot nematode) (Akhtar and Siddiqui 2008). The inoculation of *C. arietinum* with the AMF *Glomus intraradices* and PGPRs *Pseudomonas alcaligenes* and *Bacillus pumilus* reduced the combined impact of *M. phaseolina* and *M. incognita* when compared to single-strain inoculants, dual-strain inoculants and controls. This synergism serves as an indication that experiments focusing on the effects of single microbial species may overlook important multipartite interactions of more naturalistic microbial communities. The study of microbe–microbe synergism might provide valuable models to decipher underlying communication and validate these findings in more complex microbial communities.

## Conclusions

Increasing demands for food by a growing human population, along with agricultural challenges posed by climate change, are risks to global food security. Microbes in the rhizosphere are involved in many processes that determine agricultural soil productivity, including preservation of soil structure, nutrient recycling, disease control and degradation of pollutants. Agricultural practices can negatively impact soil microbes by reducing organic matter content in the soil and cause contamination of groundwater. In this context, understanding the potential for manipulation of soil microbial communities to increase crop yields and reduce losses is highly relevant. Much research has focused on the potential for individual microbial strains to bring benefits to plants and has clearly demonstrated the potential benefits to agriculture of application of microbial treatments. However, it is also clear that microbes can act synergistically to impact plant health and development, and that edaphic factors also play an important role in root microbiome formation. The development of disease suppressive soils following successive seasons of crop monoculture further suggests that stable beneficial soil microbial communities can develop and be maintained without deliberate attempts at modification by humans, and are presumably induced by conditions that provide a stable environment for plant-beneficial microbial partners. The growing body of research relating to plant–microbe interactions and their effects is bringing into focus the importance of these relationships to plant health and productivity. While our understanding of the importance of these interactions is increasing, there is still a requirement for research to unravel the intricacies of communication between all members of the root microbiome and their plant hosts. The multipartite interactions that lead to assembly and maintenance of the root microbiome are highly complex and not fully understood. A greater understanding of root microbiome community dynamics and communication has the potential to allow for more efficient exploitation of this largely untapped resource. Farming methods that support recruitment and maintenance of beneficial microbial communities in the rhizosphere could provide benefits to agriculture in the form of enhanced crop yields and disease suppression.
